# Supplementary material for: Unraveling the Impact of Environmental Factors and Evolutionary History on Species Richness Patterns of the Genus Sorbus at Global Level
Source: Plants (Basel). 2025 Jan 23;14(3):338. doi: 10.3390/plants14030338 (PMC11820190; doi:10.3390/plants14030338)

Figure S1 The divergence time of *Sorbus*. Blue bars represent 95% highest posterior credible age intervals for each node. The number above nodes is mean divergence age.

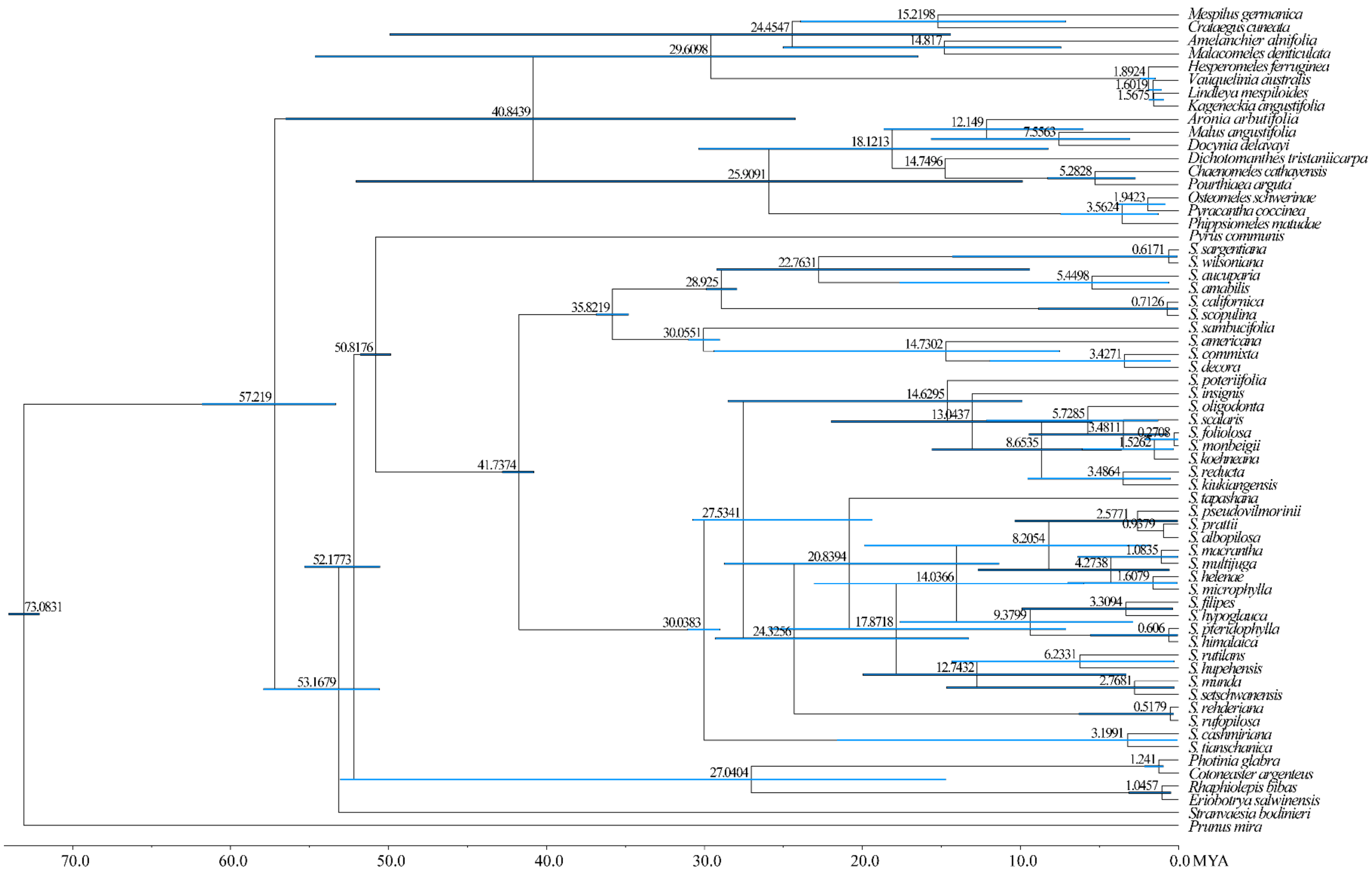

Supplement: Supplementary file 1 [file plants-14-00338-s001.zip › Figure S1.pdf]
