# Supplementary material for: Unraveling the Impact of Environmental Factors and Evolutionary History on Species Richness Patterns of the Genus Sorbus at Global Level
Source: Plants (Basel). 2025 Jan 23;14(3):338. doi: 10.3390/plants14030338 (PMC11820190; doi:10.3390/plants14030338)
Supplement: Supplementary file 1 [file plants-14-00338-s001.zip › Table S1.pdf]

Table S1. List of *Sorbus* species and distribution.

Scientific Name	Asia	Europe	North America	Hengduan Mountains
Sorbus albopilosa T.T.Yu & L.T.Lu	1	0	0	1
Sorbus amabilis W. C. Cheng ex Z. X. Yu	1	0	0	0
Sorbus americana Marshall	0	0	1	0
Sorbus amoena Mc All.	1	0	0	1
Sorbus arachnoidea Koehne	1	0	0	1
Sorbus aucuparia L.	1	1	0	0
Sorbus aucuparia subsp. *glabrata* (Wimm. & Grab.) Hedl.	1	1	0	0
Sorbus aucuparia subsp. *praemorsa* (Guss.) Nyman	0	1	0	0
Sorbus bissetii McAll.	1	0	0	1
Sorbus bulleyana McAll.	1	0	0	1
Sorbus californica Greene	0	0	1	0
Sorbus carmesina McAll.	1	0	0	1
Sorbus cashmiriana Hedl.	1	0	0	0
Sorbus cibagouensis H.Peng & Z.J.Yin	1	0	0	1
Sorbus cinereopubescens McAll	1	0	0	0
Sorbus commixta Hedl.	1	0	0	0
Sorbus commixta var. *ulleungensis* (Chin S. Chang) M. Kim	1	0	0	0
Sorbus coxii Mc All.	1	0	0	1
Sorbus decora (Sarg.) C. K. Schneid.	0	1	1	0
Sorbus discolor (Maxim.) Maxim.	1	0	0	0
Sorbus dolichofoliolatus X.F.Gao & Meng Li	1	0	0	1
Sorbus doshonglaensis Xin Chen, Xiao C. Zhang & C. Q. Tang	1	0	0	1
Sorbus eburnea Mc All.	1	0	0	1
Sorbus ellipsoidalis Mc All.	1	0	0	1
Sorbus erythrosepala Kainul.	1	0	0	1
Sorbus erzincanica Dönmez	0	1	0	0
Sorbus esserteauiana Koehne	1	0	0	1
Sorbus fansipanensis Mc All.	1	0	0	0
Sorbus filipes Hand.-Mazz.	1	0	0	1
Sorbus foliolosa (Wall.) Spach	1	0	0	1
Sorbus forrestii Mc All. & Gillham	1	0	0	1
Sorbus frutescens Mc All.	1	0	0	0
Sorbus gilgitana Mc All.	1	0	0	0
Sorbus glabriuscula Mc All.	1	0	0	1
Sorbus glomerulata Koehne	1	0	0	1
Sorbus gonggashanica Mc All.	1	0	0	1
Sorbus gongshanensis X. F. Gao & Meng Li	1	0	0	1
Sorbus gracilis (Siebold & Zucc.) K. Koch	1	0	0	0
Sorbus harrowiana (Balf. fil. & W. W. Sm.) Rehder	1	0	0	1
Sorbus helenae Koehne	1	0	0	1
Sorbus helenae var. *argutiserrata* T. T. Yu	1	0	0	1

Sorbus himalaica Gabrieljan	1	0	0	0
Sorbus hugh-mcallisteri Mikolás	1	0	0	1
Sorbus hupehensis C.K.Schneid.	1	0	0	1
Sorbus hupehensis var. *paucijuga* (D. K. Zang & P. C. Huang) L. T. Lu	1	0	0	0
Sorbus hypoglauca (Cardot) Hand.-Mazz.	1	0	0	1
Sorbus insignis (Hook. fil.) Hedl.	1	0	0	1
Sorbus keenanii Rushforth	1	0	0	0
Sorbus khumbuensis Mc All.	1	0	0	0
Sorbus kiukiangensis T.T.Yu	1	0	0	1
Sorbus kiukiangensis var. *glabrescens* T.T.Yu	1	0	0	1
Sorbus koehneana C. K. Schneid.	1	0	0	1
Sorbus kongboensis McAll.	1	0	0	0
Sorbus kurzii (G. Watt ex Prain) C. K. Schneid.	1	0	0	1
Sorbus lanpingensis L. T. Lu	1	0	0	1
Sorbus lingshiensis Rushforth	1	0	0	0
Sorbus longii Rushforth	1	0	0	0
Sorbus lushanensis Xin Chen & Jing Qiu	1	0	0	0
Sorbus macallisteri Rushforth	1	0	0	0
Sorbus macrantha Merr.	1	0	0	1
Sorbus maderensis (Lowe) Dode	0	1	0	0
Sorbus matsumurana (Makino) Koehne	1	0	0	0
Sorbus microphylla (Wall. ex Hook. fil.) Wenz.	1	0	0	1
Sorbus monbeigii (Cardot) N. P. Balakr.	1	0	0	1
Sorbus muliensis Mc All.	1	0	0	1
Sorbus multijuga Koehne	1	0	0	1
Sorbus munda Koehne	1	0	0	1
Sorbus obsoletidentata (Cardot) T. T. Yu	1	0	0	1
Sorbus occidentalis (S. Watson) Greene	0	0	1	0
Sorbus oligodonta (Cardot) Hand.-Mazz.	1	0	0	1
Sorbus olivacea Mc All.	1	0	0	1
Sorbus ovalis McAll.	1	0	0	1
Sorbus parva McAll.	1	0	0	1
Sorbus parvifructa Mc All.	1	0	0	1
Sorbus pohuashanensis (Hance) Hedl	1	0	0	0
Sorbus poteriifolia Hand.-Mazz.	1	0	0	1
Sorbus prattii Koehne	1	0	0	1
Sorbus prattii var. *aestivalis* (Koehne) T. T. Yu	1	0	0	1
Sorbus pseudohupehensis Mc All.	1	0	0	1
Sorbus pseudovilmorinii Mc All.	1	0	0	1
Sorbus pteridophylla Hand.-Mazz.	1	0	0	1
Sorbus randaiensis (Hayata) Koidz.	1	0	0	0
Sorbus reducta Diels	1	0	0	1
Sorbus reducta var. *pubescens* L. T. Lu	1	0	0	1

Sorbus rehderiana Koehne	1	0	0	1
Sorbus rehderiana var. *cupreonitens* Hand.-Mazz.	1	0	0	1
Sorbus rehderiana var. *grosseserrata* Koehne	1	0	0	1
Sorbus rinzenii Rushforth	1	0	0	0
Sorbus rosea Mc All.	1	0	0	0
Sorbus rubescens Mc All.	1	0	0	1
Sorbus rufo-ferruginea C.K. Schneider	1	0	0	0
Sorbus rufopilosa C. K. Schneid.	1	0	0	1
Sorbus rufopilosa var. *stenophylla* Koehne	1	0	0	0
Sorbus rushforthii McAll.	1	0	0	0
Sorbus rutilans Mc All.	1	0	0	1
Sorbus sambucifolia (Cham. & Schltdl.) M. Roem.	1	0	0	0
Sorbus sargentiana Koehne	1	0	0	1
Sorbus scalaris Koehne	1	0	0	1
Sorbus scopulina Greene	0	0	1	0
Sorbus setschwanensis (C. K. Schneid.) Koehne	1	0	0	1
Sorbus sitchensis M. Roem.	0	0	1	0
Sorbus splendens Grimshaw & C.R.Sanders	1	0	0	0
Sorbus tapashana C. K. Schneid.	1	0	0	0
Sorbus tenuis McAll.	1	0	0	1
Sorbus tianschanica Rupr.	1	0	0	1
Sorbus tianschanica var. *integrifoliolata* T. T. Yu	1	0	0	0
Sorbus ursina (Wall.) Decne.	1	0	0	1
Sorbus vilmorinii C. K. Schneid.	1	0	0	1
Sorbus wilsoniana C.K.Schneid.	1	0	0	1
Sorbus zayuensis T. T. Yu & L. T. Lu	1	0	0	1

Note: 0 – absent, 1 – present
