# Supplementary material for: Unraveling the Impact of Environmental Factors and Evolutionary History on Species Richness Patterns of the Genus Sorbus at Global Level
Source: Plants (Basel). 2025 Jan 23;14(3):338. doi: 10.3390/plants14030338 (PMC11820190; doi:10.3390/plants14030338)
Supplement: Supplementary file 1 [file plants-14-00338-s001.zip › Table S2.pdf]

Table S2. Accession numbers for the *Sorbus* and outgroup species used in this study.

Taxon	Accession number
Sorbus albopilosa	NC068534
Sorbus amabilis	MT357029
Sorbus americana	MZ984219
Sorbus aucuparia	MT610101
Sorbus californica	NC085651
Sorbus cashmiriana	NC085671
Sorbus commixta	MK920288
Sorbus decora	NC085652
Sorbus filipes	NC068533
Sorbus foliolosa	*
Sorbus helenae	NC068536
Sorbus himalaica	NC085572
Sorbus hupehensis	NC068721
Sorbus hypoglauca	NC068535
Sorbus insignis	NC051947
Sorbus kiukiangensis	NC085636
Sorbus koehneana	*
Sorbus macrantha	NC085631
Sorbus microphylla	NC085633
Sorbus monbeigii	*
Sorbus multijuga	NC068724
Sorbus munda	NC062714
Sorbus oligodonta	NC085634
Sorbus poteriifolia	NC085663
Sorbus prattii	MK814479
Sorbus pseudovilmorinii	NC085664
Sorbus pteridophylla	ON049651
Sorbus reducta	NC068722
Sorbus rehderiana	OK012001
Sorbus rufopilosa	NC085638
Sorbus rutilans	NC068528
Sorbus sambucifolia	NC085654
Sorbus sargentiana	NC068529
Sorbus scalaris	NC085637
Sorbus scopulina	NC085658
Sorbus setschwanensis	NC046777
Sorbus tapashana	NC085591
Sorbus tianschanica	NC068599
Sorbus wilsoniana	NC068531
Amelanchier alnifolia	MN068255
Aronia arbutifolia	MN061996
Chaenomeles cathayensis	MT561270
Cotoneaster argenteus	MK578683
Crataegus cuneata	NC058896
Dichotomanthes tristaniicarpa	MN577869

Docynia delavayi	MN506259
Eriobotrya salwinensis	NC045348
Hesperomeles ferruginea	NC045328
Kageneckia angustifolia	MN068264
Lindleya mespiloides	MN068248
Malacomeles denticulata	MN068267
Malus angustifolia	MN061984
Mespilus gemanica	MK920295
Osteomeles scherinae	MN062000
Phippsiomeles matudae	MN062002
Photinia glabra	NC062337
Pourthiaea arguta	MN061991
Prunus mira	NC040125
Pyracantha coccinea	OM232776
Pyrus communis	LT996903
Rhaphiolepis bibas	MT479167
Stranvaesia bodinieri	MK920276
Vauquelinia australis	MN068250

Note：* indicates newly measured sequences
